# A pretargeted multimodal approach for image-guided resection in a xenograft model of colorectal cancer

**DOI:** 10.1186/s13550-019-0551-4

**Published:** 2019-09-04

**Authors:** Fortuné M. K. Elekonawo, Susanne Lütje, Gerben M. Franssen, Desirée L. Bos, David M. Goldenberg, Otto C. Boerman, Mark Rijpkema

**Affiliations:** 1 Department of Radiology and Nuclear Medicine, Radboud University Medical Center, PO Box 9101, 6500 HB Nijmegen, The Netherlands; 2 Garden State Cancer Center, Center for Molecular Medicine and Immunology, Morris Plains, NJ USA

**Keywords:** Colorectal cancer, Pretargeting, Near-infrared fluorescence, Image-guided, Carcinoembryonic antigen

## Abstract

**Background:**

Image-guided surgery may improve surgical outcome for colorectal cancer patients. Here, we evaluated the feasibility of a pretargeting strategy for multimodal imaging in colorectal cancer using an anti-carcinoembryonic antigen (CEA) x anti-histamine-succinyl-glycine (HSG) bispecific antibody (TF2) in conjunction with the dual-labeled diHSG peptide (RDC018), using both a fluorophore for near-infrared fluorescence imaging and a chelator for radiolabeling.

**Methods:**

Nude mice with subcutaneous (s.c) CEA-expressing LS174T human colonic tumors and CEA-negative control tumors were injected with TF2. After 16 h, different doses of ^111^In-labeled IMP-288 (non-fluorescent) or its fluorescent derivative RDC018 were administered to compare biodistributions. MicroSPECT/CT and near-infrared fluorescence imaging were performed 2 and 24 h after injection. Next, the biodistribution of the dual-labeled humanized anti-CEA IgG antibody [^111^In]In-DTPA-hMN-14-IRDye800CW (direct targeting) was compared with the biodistribution of ^111^In-RDC018 in mice with TF2-pretargeted tumors, using fluorescence imaging and gamma counting. Lastly, mice with intraperitoneal LS174T tumors underwent near-infrared fluorescence image-guided resection combined with pre- and post-resection microSPECT/CT imaging.

**Results:**

^111^In-RDC018 showed specific tumor targeting in pretargeted CEA-positive tumors (21.9 ± 4.5 and 10.0 ± 4.7% injected activity per gram (mean ± SD %IA/g), at 2 and 24 hours post-injection (p.i.), respectively) and a biodistribution similar to ^111^In-IMP288. Both fluorescence and microSPECT/CT images confirmed preferential tumor accumulation. At post mortem dissection, intraperitoneal tumors were successfully identified and removed using pretargeting with TF2 and ^111^In-RDC018.

**Conclusion:**

A pretargeted approach for multimodal image-guided resection of colorectal cancer in a preclinical xenograft model is feasible, enables preoperative SPECT/CT, and might facilitate intraoperative fluorescence imaging.

**Electronic supplementary material:**

The online version of this article (10.1186/s13550-019-0551-4) contains supplementary material, which is available to authorized users.

## Introduction

Colorectal cancer is the third most common cause of cancer deaths in the Western world [1]. In different stages of disease, surgery is a crucial part of the (curative) treatment of patients and complete resection of malignant tissue remains one of the main prognostic factors [2]. Surgical outcome may be improved by better pre- and intraoperative imaging tools to aid the surgeon in patient selection, tumor detection, and radical resection. Intraoperative fluorescence imaging has shown potential to increase specificity and sensitivity of resections [3]. A combined approach to improve both pre- and intraoperative tumor detection using tumor-targeted multimodal imaging may be advantageous to achieve the best surgical outcome.

Overexpression of carcinoembryonic antigen (CEA) is present in 90–95% colorectal cancers [4, 5]. This biomarker may be targeted by the high-affinity monoclonal antibody hMN-14. hMN-14 is a humanized IgG directed against the carcinoembryonic antigen-related cell adhesion molecule 5 [6]. In a previous study, hMN-14 labeled with Indium-111 (^111^In) and conjugated to IRDye800CW ([^111^In]In-DTPA-hMN-14-IRDye800CW) was shown to specifically accumulate in CEA-expressing tumor xenografts and enabled radio- and fluorescence-guided surgery of colorectal tumor nodules [7]. However, due to the slow blood clearance of antibodies, high tumor-to-background signals can only be achieved at several days after injection.

An alternative approach to deliver radioactive, fluorescent, or other agents to tumors for imaging and therapy is via a pretargeting strategy [8]. In this multistep approach, first a bispecific antibody (bsAb) is administered that specifically targets the tumor. Subsequently, a hapten, carrying a diagnostic or therapeutic load, is administered that binds to the bsAb. This hapten is a relatively small molecule with rapid renal clearance. Both high specificity and high tumor-to-background ratios may be achieved using this strategy, as it combines the specific targeting properties of antibodies with the favorable pharmacokinetics and clearance of small molecules [9–14].

The current study investigates the use of a tumor-specific multimodal pretargeting strategy. For this purpose, we apply the trivalent bispecific anti-CEA x anti-histamine-succinyl-glycine (HSG) antibody TF2 in combination with the multimodal di-HSG hapten-peptide RDC018, an IMP-288 derivative (Additional file 1: Figure S1). RDC018 contains both a fluorophore and a chelator for ^111^In labeling, thus enabling both radionuclide and fluorescence imaging, whereas IMP-288 only harbors a chelator and no fluorophore. Here, we evaluate the feasibility of this multimodal pretargeting approach in a colorectal cancer model of peritoneal metastasis.

## Methods

### Pretargeting molecules TF2 and RDC018

The bsAb TF2 contains two CEACAM5 and one HSG-binding sites [13] and was produced using the Dock-and-Lock method as described previously [15]. A schematic representation of the pretargeting agents is provided by Schoffelen et al. [13]. RDC018 is a peptide-hapten derived from IMP-288, a 1,4,7,10-tetraazacyclododecane-1,4,7,10-tetraacetic acid (DOTA)-conjugated d-Tyr-d-Lys-d-Glu-d-Lys tetrapeptide, in which both lysine residues are substituted with a HSG moiety via their ε-amino group (Additional file 1: Figure S1) [16]. In addition to the DOTA chelate for radiolabeling, RDC018 is C-terminally conjugated with the fluorescent DyLight^TM^ 800 NHS ester. TF2 [15] and RDC018 were kind gifts from Immunomedics Inc.

### Radiolabeling

All labeling procedures were performed under metal-free conditions. Briefly, [^111^In]InCl_3_ (Mallinckrodt Medical BV/Curium, Petten, the Netherlands) was added to IMP-288 or RDC018 in two volumes of 0.1 M 2-(*N*-morpholino)ethanesulfonic acid (MES), pH 5.5. After 20 min of incubation at 95 °C, 50 mM ethylenediaminetetraacetic acid (EDTA) was added to the labeling reaction to a final concentration of 1 mM EDTA to chelate unincorporated ^111^In. Lastly, Tween80 (Sigma-Aldrich, Saint Louis, MO, USA) was added to the labeling product in a final concentration of 0.01%. The labeling efficiency was determined by instant thin-layer chromatography on Varian silica gel strips (ITLC-SG; Agilent Technologies, Amstelveen, the Netherlands) using 0.1 mM ammonium acetate (NH_4_Ac) buffer with 0.1 M EDTA (pH 5.5) as the mobile phase. If labeling efficiency was below 95%, labeled products were purified using solid-phase extraction on an HLB cartridge (Waters Chromatography B.V., Etten-Leur, the Netherlands) with 100% EtOH as mobile phase. Final radiochemical purity was > 95% for all compounds.

The antibody hMN-14 was conjugated to IRDye800CW (fluorophore:antibody substitution ratio 1.4) and diethylenetriaminepentaacetic acid (DTPA) which was labeled with [^111^In]InCl_3_ at a specific activity of 0.78 MBq μg^−1^, as previously described [7].

### Cell culture

CEA-expressing human colon adenocarcinoma cells LS174T and CEA-negative human renal cell carcinoma cells SK-RC-52 were obtained from the American Type Culture Collection (ATCC, Manassas, VA, USA). Cells were tested for mycoplasma negativity. Cells were cultured in sterile conditions using RPMI-1640 medium supplemented with 10% heat-inactivated fetal calf serum and 2 mM l-glutamine, without antibiotic additive. Cells were cultured in T150 tissue culture flasks in a humidified incubator with an atmosphere of 95% air and 5% carbon dioxide at 37 °C. All cells were harvested with trypsin/EDTA.

### Xenograft mouse models

All animal experiments were approved by the Institutional Animal Welfare Committee of the Radboud University Medical Center and were conducted in accordance to the guidelines of the Revised Dutch Act on Animal Experimentation (2014).

Female BALB/cAnNRj-*Foxn1*^*nu/nu*^ nude mice (7–9 weeks-old, 18–22-g body weight; Janvier), housed in individually ventilated cages (5 mice per cage) under non-sterile standard conditions with free access to standard animal chow and water, were adapted to laboratory conditions for 1 week before experimental use. For the biodistribution and microSPECT/CT experiments, mice were subcutaneously inoculated with 2 × 10^6^ LS174T cells (left flank) and 2 × 10^6^ SK-RC-52 cells (right flank) both suspended in 200 μL RPMI-1640 medium. For the image-guided resection experiment, intraperitoneal tumor growth was induced by an intraperitoneal injection of 3 × 10^5^ LS174T cells suspended in 200 μL RPMI-1640 medium. Tail vein injections were performed for intravenous administration of antibodies and peptides.

### Biodistribution studies

#### Biodistribution of ^111^In-IMP-288 versus ^111^In-RDC018

In the first experiment, the biodistributions of ^111^In-IMP-288 and ^111^In-RDC018 were compared. Three different dose levels (0, 0.8, and 8 nmol) of TF2 or controls were tested with a 1:20 TF2:HSG-peptide ratio of each HSG-peptide in two subsets of 30 mice (5 mice per group). Mice at the zero dose level received the same amount of HSG-peptide (0.4 nmol) as mice at the highest dose level. Seventeen days after tumor inoculation, 200 μL TF2 in PBS-0.5% BSA or PBS-0.5% BSA was injected intravenously. Sixteen hours following TF2 administration, the radiolabeled HSG peptide (^111^In-IMP-288, 9.5 MBq μg^−1^ or ^111^In-RDC018, 5.8 MBq μg^−1^) was injected. Mice were euthanized by CO_2_/O_2_ asphyxiation, and the biodistribution of ^111^In-IMP-288 and ^111^In-RDC018 was determined 2 or 24 h after peptide injection. For this purpose, tissues of interest (tumor, muscle, lung, spleen, kidney, liver, pancreas, stomach, and duodenum) were dissected and weighed after which activity was measured in a shielded 3-in.-well-type γ-counter (Perkin-Elmer, Boston, MA, USA). Blood samples were obtained by heart puncture. For calculation of the uptake of activity in each tissue as a fraction of the injected activity, three aliquots of the injection dose were counted in the γ-counter simultaneously.

#### Biodistribution of ^111^In-RDC018 versus dual-labeled hMN-14

In the second experiment, the biodistribution profile of ^111^In-RDC018 in the pretargeted approach (TF2-RDC018) was compared to the dual-labeled humanized monoclonal antibody hMN-14 (reference compound) using the IVIS Lumina fluorescence camera (Xenogen VivoVision IVIS Lumina II, Caliper Life Sciences, Waltham, MA, USA) and MicroSPECT/CT (U-SPECT II; MILabs, Utrecht, the Netherlands). TF2 (1.4 nmol) and [^111^In]In-DTPA-hMN-14-IRdye800CW (32.2 μg, 0.78 MBq μg^−1^) were injected intravenously 17 days following subcutaneous tumor cell inoculation in two groups of 5 mice. Radiolabeled RDC018 (126 MBq μg^−1^, 0.18 μg per mouse, 22 MBq per mouse) was administered 16 h following TF2 injection. Mice which received TF2 and ^111^In-RDC018 were imaged at two time points (2 and 24 h post administration of the radiolabeled peptide). The reference group (*n* = 5) was imaged 24 and 48 h after dual-labeled hMN-14 injection.

### MicroSPECT/CT and near-infrared fluorescence (NIRF) imaging

Mice with one CEA-positive and one CEA-negative tumor were scanned on a small-animal microSPECT/CT scanner with a 1.0-mm diameter pinhole collimator tube (acquisition time, 2 × 15 min) in prone position, followed by a CT scan (spatial resolution, 160 μm; 65 kV; 612 μA) for anatomical reference.

MicroSPECT/CT scans were reconstructed with MILabs reconstruction software, which uses an ordered-subset expectation maximalization algorithm.

NIRF images were acquired on the IVIS fluorescence imaging system (acquisition time, 5 min; binning, medium; Fstop, 2; excitation, 745 nm; excitation autofluorescence, 675 nm; emission, ICG; lamp level, high; FOV, D).

### Image-guided (post mortem) resection

After the biodistribution experiments, an image-guided resection experiment was performed. Intraperitoneal tumors were induced in 3 groups of mice and after 3 weeks the targeting agents were administered. In the first group, 6 nmol of TF2 was administered to 5 mice and 16 h following TF2 injection, 0.3 nmol of radiolabeled RDC018 was administered. Two hours following ^111^In-RDC018 injection, mice were imaged with microSPECT/CT and NIRF imaging. Hereafter, image-guided resection using the IVIS fluorescence imaging system was performed. To confirm complete resection, additional optical imaging and SPECT/CT images were acquired. Next, animals were dissected to determine the biodistribution of the radiolabeled peptide as described above.

The two remaining groups of mice served as controls. In two mice, peritoneal tumors grew faster than expected and reached a humane endpoint before the start of the experiment. These mice were therefore euthanized prior to injection of the control compounds. As a result, each control group consisted of 4 mice. In the first group, (positive control) dual-labeled hMN-14 [7] was administered to 4 mice with intraperitoneal LS174T tumors. Resection and analysis were performed 3 days after dual-labeled hMN-14 injection. As negative control, we used 4 mice with tumors pretargeted with the trivalent anti-CD20 bsAb TF4 [17] in combination with ^111^In-RDC018 with the same dosing and timing as the TF2 group.

### Statistical analysis

Statistical analyses were performed using GraphPad Prism software (version 5.03; GraphPad Software). Student’s *t* test was performed on the biodistribution studies IMP288 versus RDC018 (tumor, blood, and kidney), corrected for multiple testing (Bonferroni). A *p* value < 0.05 was used to reject the null hypothesis. Data are presented as mean and standard deviation.

## Results

### Biodistribution

**Fig. 1:**
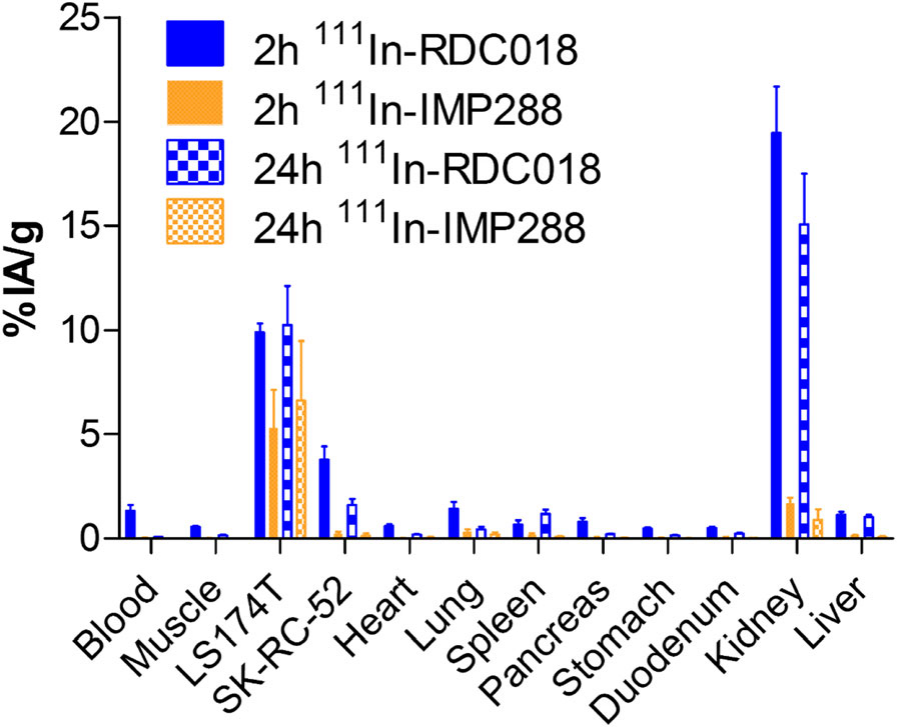
Biodistribution profiles of ^111^In-RDC018 and ^111^In-IMP-288 at 2 h and 24 h p.i. after pretargeting with 8 nmol TF2 in BALB/c nude mice, showing specific tumor uptake in the s.c. CEA-expressing LS174T tumor and pronounced renal uptake

To gain more insight into differences in the in vivo behavior between ^111^In-RDC018 and ^111^In-IMP-288, mice with subcutaneous tumors received different dose levels of TF2 and hapten-peptide. The biodistribution of ^111^In-RDC018 revealed high and target-specific uptake in CEA-expressing TF2-pretargeted tumors after 2 and 24 h (22.0 ± 4.5 %IA/g and 10.0 ± 4.7 %IA/g, respectively). In contrast, uptake of ^111^In-RDC018 remained low in CEA-negative tumors (6.0 ± 1.8 %IA/g and 0.9 ± 0.4 %IA/g, respectively) as well as in other healthy tissues. Compared to the reference compound ^111^In-IMP-288, ^111^In-RDC018 showed similar tumor-specific uptake in CEA-positive TF2-pretargeted tumors (Fig. 1, 8 nmol; TF2, Additional file 2: Figure S2, 0 nmol and 0.8 nmol TF2). However, in CEA-negative tumors, uptake of ^111^In-RDC018 was significantly higher than the uptake of ^111^In-DOTA-IMP-288 (0.13 ± 0 %IA/g and 0.07 ± 0 %IA/g *p* < 0.001). The same observation was made for tracer accumulation in the kidney and blood, which was significantly higher for ^111^In-RDC018 compared to ^111^In-IMP-288 after 2 and 24 h (both *p* < 0.001). For 0.8 nmol TF2, tumor uptake of pretargeted LS174T tumors was significantly higher compared to the negative control tumors for both time points. In more detail, for 0.8 nmol TF2 and 2 h after peptide injection, the uptake in CEA-positive LS174T tumors was 22.0 ± 4.5 %IA/g and 6.0 ± 1.8 %IA/g in CEA-negative SK-RC-52 tumors (*p* < 0.001). This difference remained 24 h after peptide injection (10.0 ± 4.7 %IA/g and 0.9 ± 0.4 %IA/g (*p* < 0.001), respectively). We did not observe a statistically significant difference in uptake between ^111^In-RDC018 and ^111^In-IMP-288 for both time points. For 8.0 nmol TF2, we observed similar results (Fig. 1). Two hours after peptide injection, uptake was 9.9 ± 0.4 %IA/g for LS174T and 3.8 ± 0.6 %IA/g for SK-RC-52 cells (*p* < 0.001). This difference remained 24 h after peptide injection (10.2 ± 1.9 %IA/g and1.6 ± 0.3 %IA/g (*p* < 0.001), respectively). Two hours after peptide injection, ^111^In-RDC018 uptake in LS174T tumors was higher than ^111^In-IMP-288: 9.9 ± 0.4 %IA/g and 5.3 ± 1.8 %IA/g (*p* = 0.0135). After 24 h, however, the uptake of ^111^In-RDC018 and ^111^In-IMP-288 was not statistically significantly different (10.2 ± 1.9 and 6.6 ± 2.9 (*p* = 0.2713).

**Fig. 2:**
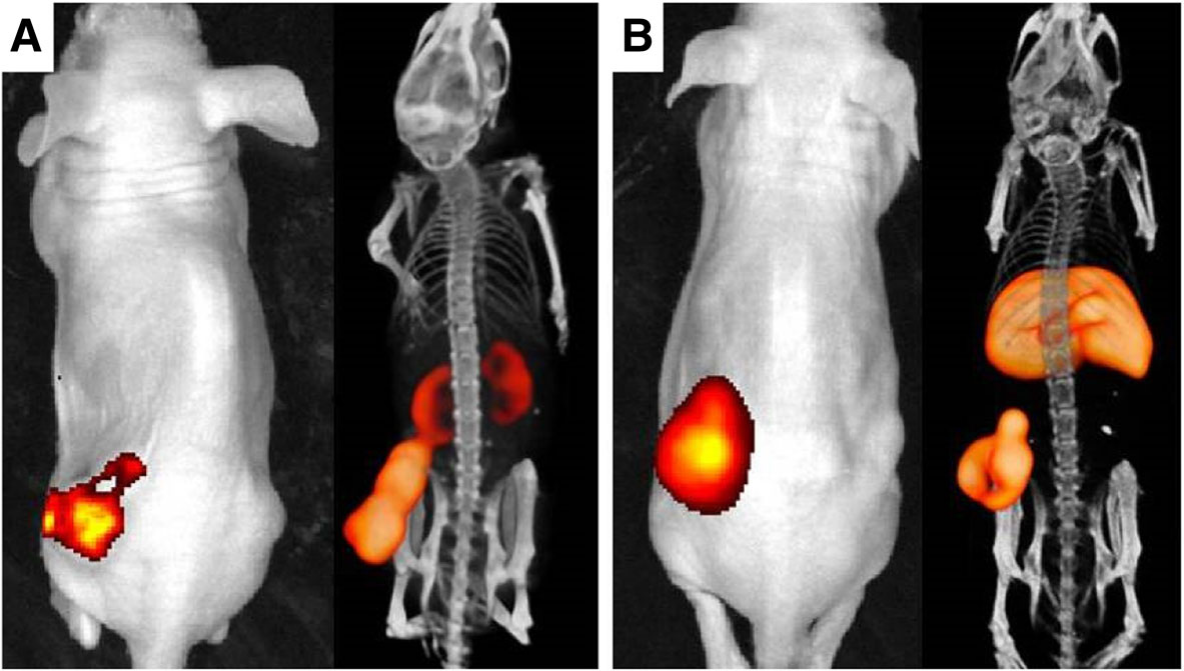
Near-infrared fluorescence (left) and microSPECT/CT (right) images of mice bearing s.c. CEA-expressing tumors (left flank), acquired using the TF2-^111^In-RDC018 pretargeting strategy (**a**) or the dual-labeled hMN-14 direct targeting strategy (**b**). Both series were acquired 24 h post injection. Note the uptake in the tumor, liver, and kidneys depending on tracer type

To compare the in vivo behavior of ^111^In-RDC018 in TF2-pretargeted tumors to dual-labeled hMN-14 (direct targeting), the biodistribution patterns of both approaches were characterized. Both tracers were shown to specifically target the CEA-expressing tumors which was confirmed by microSPECT/CT and NIRF imaging (Fig. 2). Also, the excretion routes of ^111^In-RDC018 (via the kidneys) and ^111^In-hMN-14-IRDye800CW (via the liver) was clearly illustrated (Fig. 2).

### Fluorescence imaging

**Fig. 3:**
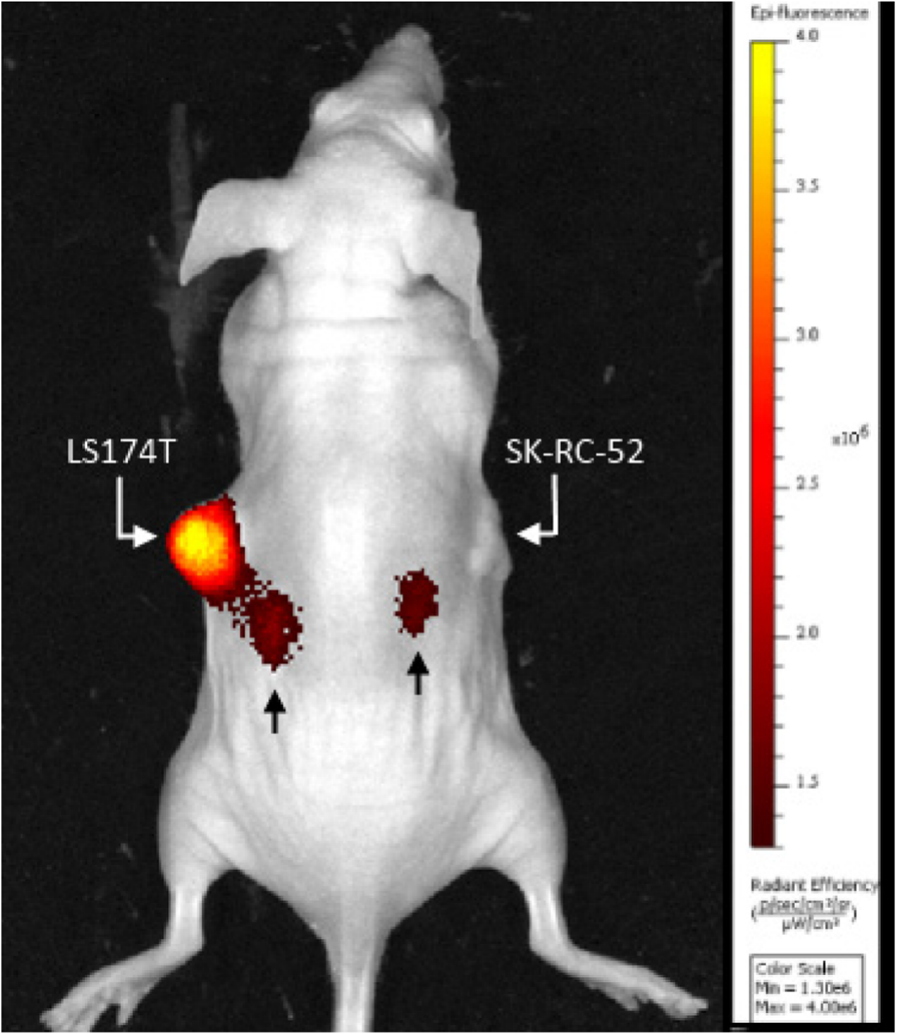
Near-infrared fluorescence image 24 h p.i. of ^111^In-RDC018 in a mouse bearing a s.c. CEA-expressing LS174T tumor (left flank) and a s.c. CEA-negative SK-RC-52 tumor (right flank), confirming the specific tumor targeting in the CEA-expressing tumor and renal clearance (black arrows) of ^111^In-RDC018

In addition to the quantitative biodistribution studies based on the radiobsignal, we assessed tumor accumulation and distribution of the tracer by NIRF imaging. The imaging results show a similar distribution pattern compared to the quantitative biodistributions (Fig. 3), demonstrating that our pretargeted approach can be used for NIRF imaging of CEA-positive tumors, which is essential for reliable image-guided surgery.

### Image-guided (post mortem) resection

**Fig. 4:**
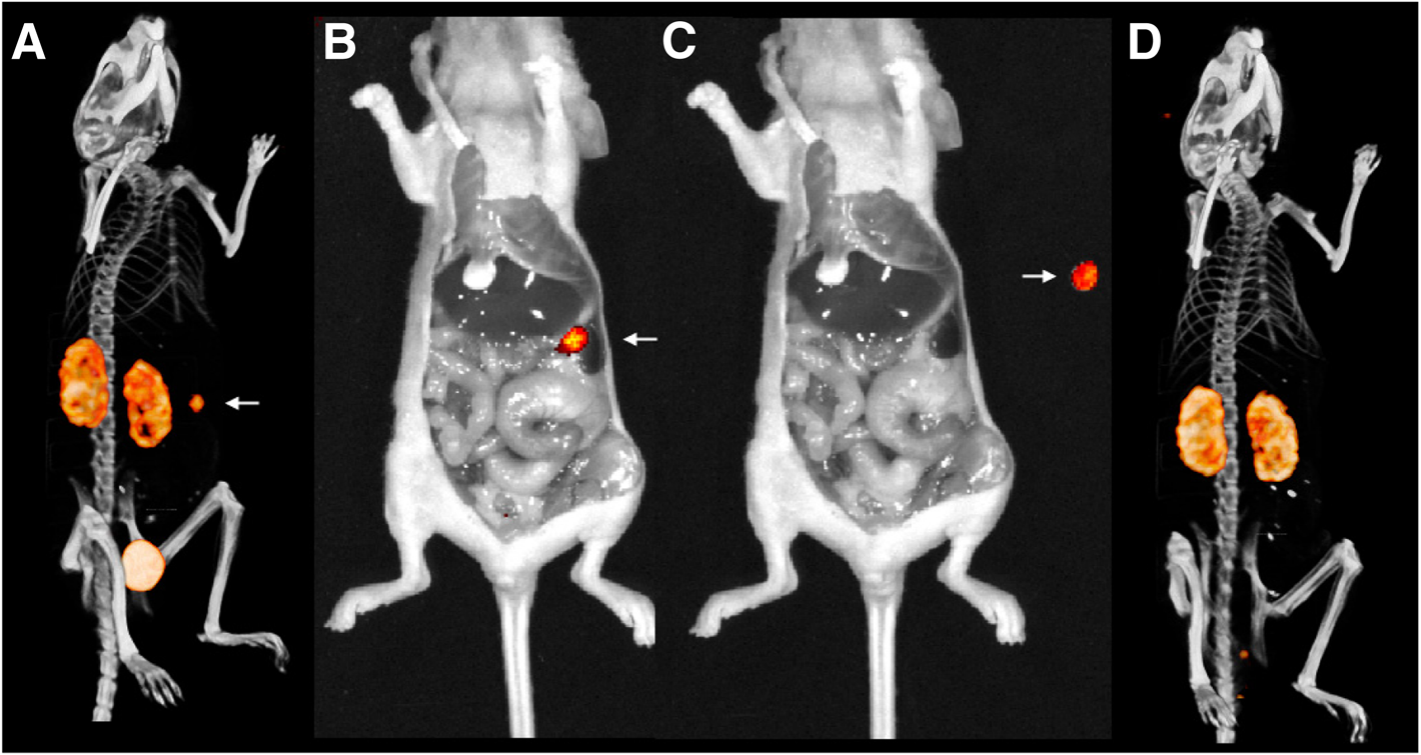
Near-infrared fluorescence images (**b**, **c**) and microSPECT/CT images (**a**, **d**) of a mouse with a CEA-expressing intraperitoneal LS174T tumor (arrow) 2 h after administration of ^111^In-RDC018. Pre-resection, the tumor can be clearly localized using microSPECT/CT (**a**, white arrow) and near-infrared fluorescence imaging (**b**, white arrow). Subsequently, after euthanization, the tumor was resected with fluorescence image guidance (**c**, white arrow). Finally, a post-resection microSPECT/CT was acquired (**d**) confirming the complete resection of the tumor nodule. The radio signal of the renal clearance of ^111^In-RDC018 can also be observed in **a** and **d**

Finally, we assessed the feasibility for pretargeted image-guided surgery in a more clinically relevant setting in mice with intraperitoneal LS174T tumors undergoing resection. RDC018 was labeled at a specific activity of 20.1 MBq μg^−1^. The microSPECT/CT images clearly identified intraperitoneal tumors (Fig. 4). Subsequently, NIRF imaging was able to identify these tumors and serve as guidance during (post mortem) resection. Finally, post-resection microSPECT/CT and NIRF imaging confirmed complete resection of tumor tissue (Fig. 4).

## Discussion

In the present study, we show that pretargeted multimodal image-guided resection is feasible in a model for peritoneal metastasis. ^111^In-RDC018 accumulation in the tumor is specific and RDC018 clears via the kidneys in TF2-pretargeted intraperitoneally xenografted tumors. These characteristics enabled “preoperative” microSPECT and “intraoperative” NIRF imaging for successful image-guided post mortem resection and confirmation of complete tumor resection by postoperative SPECT.

For the pretargeting strategy to work, the bispecific antibody should not show fast internalization. TF2 internalizes only minimally [18]. Furthermore, Schmidt et al. showed that CEA is a slowly and minimally internalizing antigen [19]. RDC018 is an IMP-288 derived hapten-peptide conjugated with a fluorescent moiety. To evaluate the effect of the fluorophore of RDC018 on the in vivo behavior of the molecule, the biodistribution was compared to that of IMP-288. Our results illustrate that ^111^In-RDC018 and ^111^In-IMP-288 have, apart from uptake in the CEA-negative tumor and kidneys, similar distribution patterns after TF2 pretargeting (Fig. 1). However, there is more nonspecific uptake of ^111^In-RDC018 across all included organs. These findings suggest that the fluorophore does not relevantly compromise the parental molecule’s binding properties and in vivo behavior. We did, however, find a higher uptake of ^111^In-RDC018 in the CEA-negative SK-RC-52 tumors (Fig. 1), which indicates more nonspecific uptake of the tracer, possibly due to different molecular characteristics of the dye (e.g., lipophilicity and charge) and/or the enhanced permeability and retention effect due to the longer blood retention time [20]. An earlier study indicated that high dye:antibody conjugation ratios (> 2) can significantly change biodistributions of antibody-dye conjugates [21]. Therefore, the smaller molecule ^111^In-RDC018 contains a single fluorescent moiety and hMN-14 was conjugated at a final dye:antibody ratio of 1.4. Further evaluation of chemical differences was beyond the scope of this study. The uptake of ^111^In-RDC018 in CEA-positive tumors, however, was large enough to provide sufficient tumor-specific uptake for imaging purposes (Figs. 2, 3, and 4). Earlier studies already showed that a non-CEA-specific pretargeting IgG combined with a radiolabeled hapten resulted in very low tumor uptake [18]. Kidney uptake was higher for ^111^In-RDC018 than for ^111^In-IMP-288, indicating enhanced tubular reabsorption of the dual-labeled peptide in the kidneys, most probably caused by charge of the fluorescent moiety.

Sharkey et al. and Lütje et al. demonstrated a pretargeted approach for radioimmunotherapy and image-guided surgery in a model of prostate cancer [10, 17]. Similar to their studies, we found high specific tumor uptake and higher kidney uptake when we injected different doses of the bispecific antibody TF2 and the ^111^In-labeled diHSG hapten-peptide RDC018. High renal uptake of ^111^In-RDC018 as a result of renal clearance might impair imaging and image guidance in the vicinity of the kidneys and bladder. This would, however, not reduce its potential in colorectal cancer, since renal involvement or dissemination is rare [22]. On the contrary, renal clearance may be advantageous for tracers targeting colorectal cancer. For example, the dual-labeled humanized antibody hMN-14 is mainly cleared via the hepatobiliary route, which results in a relatively high fluorescence and radionuclide signal in the liver [7]. Therefore, intraoperative detection of liver metastasis or peritoneal metastasis present on the visceral peritoneum of the liver may remain particularly challenging with direct targeting strategies using antibodies. Figure 1 indicates higher liver uptake for ^111^In-RDC018 than ^111^In-IMP-288. The tumor-to-liver ratio, however, is still greater than 5. In addition, the more favorable pharmacokinetics of smaller molecules involved in pretargeting may result in higher tumor-to-background ratios at early time points after injection and might render them more suitable for theranostic purposes compared to antibody-based strategies [8].

Several challenges remain before successful clinical translation of this type of pretargeting strategies, including optimizing dosing and timing, for example, the protein dose of the antibody, dose and activity of the hapten, time interval between antibody and hapten administration, and interval between hapten administration and imaging or surgery. Despite these challenges, several clinical trials that show the safety and feasibility of TF2 bsAb and hapten pretargeting strategies have been concluded [13, 23]. Recently, Liu published a more quantitative description of the pretargeting concept which contributes to overcoming the challenges of clinical translation [24]. For fluorescence image-guided surgery, also, translation of the preclinical setup to the clinical setup at the operating rooms might be challenging.

For colorectal cancer patients, the cornerstone in surgical treatment is complete and radical resection. A surgeon’s ability to distinguish benign from malignant tissue can be hampered by adhesions and fibrous or scar tissue, which may be present due to earlier intra-abdominal procedures or disease. Reliable assessment of tumor burden can therefore sometimes only be performed during the surgical procedure [25]. Imaging techniques might aid in improving patient selection and the surgeon’s ability to distinguish between benign and malignant [3]. Recently, CEA-based NIRF image-guided surgery was shown to be safe and feasible in a clinical trial with pancreatic cancer patients [26]. The same tracer was used in a trial in colorectal cancer patients with recurrent disease or peritoneal metastasis [27]. These results indicate that CEA-targeted NIRF image-guided surgery may aid the surgeon in clinical decision-making during surgery; however, the specificity found in these trials was 62%. Fluorescence has a limited penetration depth and the occurrence of false-negative tumor lesions in the former trials was mainly caused by overlying blood or tissue. Adding a radiotracer for targeted multimodality imaging can serve a multipurpose goal [14, 28]. It can serve as a preoperative detection tool (e.g., SPECT or PET) of primary tumor and/or (peritoneal) metastases [13]. Additionally, intraoperative detection of lesions using a gamma probe could be applied for deeper lesions and can potentially increase specificity and sensitivity. Furthermore, intraoperative fluorescence imaging may identify tumor lesions and might even be used as a postoperative evaluation tool for complete removal. Another advantage of adding a radiotracer to the fluorescent targeting molecules is the ability to reliably quantify the amount of tracer present in surgical specimens. Standardization in quantification of fluorescent imaging is gaining more interest [29] and could benefit from quantitative techniques using radiotracers.

## Conclusions

Our findings elucidate a potential role of pretargeting strategies in the search of optimum vehicles for image-guided surgery and theranostic approaches in modern treatment of colorectal cancer. In the current study, resection of pretargeted tumors with radio and fluorescence guidance in a colorectal cancer model was shown to be feasible. A limitation of our approach is the post mortem dissection. Therefore, the next step is optimization of this strategy before clinical translation in the future.

## Additional files

Additional file 1: **Figure S1.** Structural formulas of IMP-288 and RDC018. Blue: DOTA chelate. Red: DyLight^TM^ 800 (DOCX 48 kb)

Additional file 2: **Figure S2.** Biodistribution profiles of 111In-RDC018 and 111In-IMP-288 at 2 h and 24 h p.i. after pretargeting with 0 and 0.8 nmole TF2 in BALB/c nude mice with s.c. tumors (DOCX 156 kb)

## Data Availability

The datasets used and/or analyzed during the current study are available from the corresponding author on reasonable request.

## Abbreviations

BsAb: Bispecific antibody
CEA: Carcinoembryonic antigen
DTPA: Diethylenetriaminepentaacetic acid
HSG: Histamine-succinyl-glycine
IMP-288: Di-histamine-succinul-glycine hapten peptide
PET: Positron emission tomography
RDC018: A DyLight^TM^ 800 NHS ester-conjugated derivative of IMP-288
SPECT: Single-photon emission computed tomography
TF2: Bispecific antibody directed against CEA and HSG
