# Supplementary material for: A pretargeted multimodal approach for image-guided resection in a xenograft model of colorectal cancer
Source: EJNMMI Res. 2019 Sep 4;9:86. doi: 10.1186/s13550-019-0551-4 (PMC6726731; doi:10.1186/s13550-019-0551-4)
Supplement: Supplementary file 1 — Figure S1. Structural formulas of IMP-288 and RDC018. Blue: DOTA chelate. Red: DyLightTM 800 (DOCX 48 kb) [file 13550_2019_551_MOESM1_ESM.docx]

**IMP-288**

**RDC018**

**Figure S1.** Structural formulas of IMP-288 and RDC018. Blue: DOTA chelate. Red: DyLight^TM^ 800.
