# Supplementary material for: A pretargeted multimodal approach for image-guided resection in a xenograft model of colorectal cancer
Source: EJNMMI Res. 2019 Sep 4;9:86. doi: 10.1186/s13550-019-0551-4 (PMC6726731; doi:10.1186/s13550-019-0551-4)
Supplement: Supplementary file 2 — Figure S2. Biodistribution profiles of 111In-RDC018 and 111In-IMP-288 at 2 h and 24 h p.i. after pretargeting with 0 and 0.8 nmole TF2 in BALB/c nude mice with s.c. tumors (DOCX 156 kb) [file 13550_2019_551_MOESM2_ESM.docx]

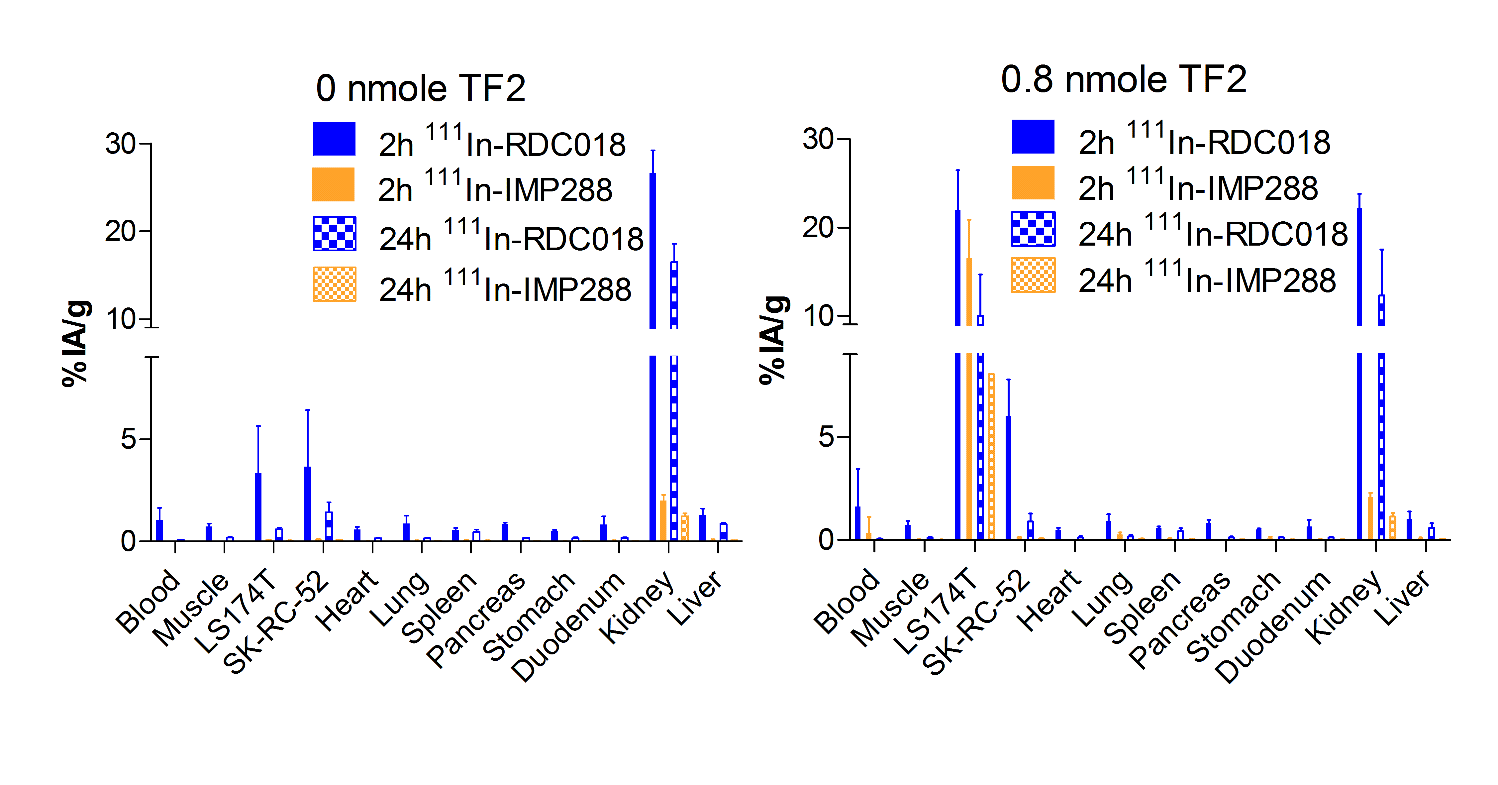


**Figure S2.** Biodistribution profiles of ^111^In-RDC018 and ^111^In-IMP-288 at 2 h and 24 h p.i. after pretargeting with 0 and 0.8 nmole TF2 in BALB/c nude mice with s.c. tumors.
